# Differences in Chemical Composition, Polyphenol Compounds, Antioxidant Activity, and In Vitro Rumen Fermentation among Sorghum Stalks

**DOI:** 10.3390/ani14030415

**Published:** 2024-01-27

**Authors:** Xingzhou Tian, Jixiao Qin, Qingyuan Luo, Yiqing Xu, Shuanglong Xie, Rui Chen, Xu Wang, Qi Lu

**Affiliations:** Key Laboratory of Animal Genetics, Breeding and Reproduction in the Plateau Mountainous Region, Ministry of Education, College of Animal Science, Guizhou University, Guiyang 550025, China; tianxingzhou@yeah.net (X.T.); zrz2942037436@163.com (J.Q.); natty579@163.com (Q.L.); tacool1001@163.com (Y.X.); xsljty@163.com (S.X.); chenruisc@163.com (R.C.); wangxu10216610@163.com (X.W.)

**Keywords:** sorghum stalk, polyphenol compound, antioxidant activity, in vitro gas production

## Abstract

**Simple Summary:**

Polyphenol compounds are important physiological activity materials that resist corrosion and oxidation, can be maintained in a state of freshness, and can be used safely, among other advantages. In China, the most important cereal crop is sorghum, which is an important raw material for brewing. However, sorghum stalks are usually considered to have no value and are thrown away or burned, resulting in environmental pollution and the possible waste of a usable resource. Notably, the sorghum stalk contains an abundance of polyphenols that exhibit great development potential as unconventional resources. However, studies on sorghum stalks with respect to their polyphenol compounds and their effects on in vitro rumen fermentation in goats have been relatively rare to date. Accordingly, in the present study, six types of sorghum (*Sorghum bicolor* L. Moench) varieties used for brewing traditional Chinese liquor were selected, and we compared their stalks in terms of their chemical composition, polyphenol compounds, antioxidant activity, and effects on in vitro rumen fermentation in goats. Our findings provide new details regarding the use of this unconventional feed for goats, as well as theories for developing this kind of resource for use as ruminant feed.

**Abstract:**

The aim of the study was to examine the differences in the chemical composition, polyphenol compounds, antioxidant activity, and in vitro rumen fermentation among six varieties of sorghum stalks. The results show that maoliangnuo 1 (M1) contained a higher (*p* < 0.05) level of dry matter, and jinzhong 405 (J4) contained a higher (*p* < 0.05) level of crude protein content. The concentrations of neutral detergent fiber, acid detergent fiber, and cellulose were significantly higher (*p* < 0.05) in stalk jinliangnuo (JN). The levels of chlorogenic acid, homoorientin, isovitexin, vitexin, rhoifolin, genistin, quercetin, apigenin, aloe emodin, emodin, and total polyphenols were all significantly (*p* < 0.05) higher in maohongnuo 6 (M6) than in the other stalks. Moreover, stalk M6 contained higher (*p* < 0.05) levels of total antioxidant capacity (TAC), glutathione peroxidase (GPX), catalase (CAT), and 2,2-diphenyl-1-picrylhydrazyl (DPPH) free-radical scavenging capacity. There were significant (*p* < 0.05) positive correlations between total polyphenols and TAC, superoxide dismutase, GPX, CAT, and DPPH free-radical scavenging capacity. The total gas production was significantly (*p* < 0.05) influenced by the sorghum stalk variety and incubation time. Stalk J4 displayed higher values for the (*p* < 0.05) immediately soluble fraction and the potential extent of gas production, while stalk M6 exhibited a significantly lower (*p* < 0.05) insoluble fraction level. Furthermore, stalk M6 exhibited a significantly higher level of (*p* < 0.05) ruminal fluid propionic acid, but its level of butyric acid and its ratio of acetic acid to propionic acid were both significantly lower (*p* < 0.05). Taken together, the results reported in this paper indicate that the chemical composition, polyphenol compounds, antioxidant activity, and in vitro rumen fermentation all vary greatly among different varieties of sorghum stalks.

## 1. Introduction

Polyphenols are important secondary metabolites in plants. They are present in plants of various colors, and exhibit strong antioxidant activities due to their unique molecular structure [1]. Indeed, polyphenol compounds have a special structure, which is characterized by hydroxyl groups on aromatic rings [2]. They mainly act to remove reactive species of chlorine, nitrogen, and oxygen, or to chelate metal ions in the initial and propagation stages of the oxidation process, resulting in increased antioxidant activity [2]. Moreover, polyphenols may promote the transfer of hydrogen atoms from the active hydroxyl group to the free radicals, indicating an antioxidant effect [3]. An increasing number of studies have shown that polyphenols exhibit a variety of biological activities and can positively impact ruminant health. For example, Aderao et al. [4] showed that polyphenol-rich plants could result in lower acetate:propionate ratios and reduced methane production, and thus enhance green livestock production. Similarly, Cattani et al. [5] suggested that polyphenols could induce a shift in the partition of energy, and stimulate microbial growth, in ruminal fluid in vitro.

Today, the full utilization of existing feed resources and the increased development of unconventional feeds both contribute to the sustainable development of the feeding industry [6]. In this regard, it is noteworthy that plants are rich in bioactive compounds which play very important roles in protecting against the effects of free radicals in ruminants [7]. Various studies have demonstrated that crop stalks could be utilized as safe natural antioxidants for animals, because they contain high amounts of natural antioxidants such as polyphenol compounds, and exhibit strong antioxidant activity [8,9]. Thus, the use of stalk polyphenol to improve antioxidant activity in animals may be an effective way to promote sustainable development in agriculture.

As the liquor capital of China, Guizhou province is famed across the world for its traditional Chinese beverages, including Moutai liquor [10]. In China, sorghum is the main raw material used for brewing traditional liquor; the level of production is high, and planting areas are extensive [11]. However, sorghum stalks are usually considered to have no value, and are thrown away or burned, resulting in environmental pollution and the possible waste of a usable resource. Notably, sorghum stalks contain abundant fibers such as hemicelluloses and cellulose, which can stimulate gastrointestinal peristalsis, maintain normal digestive function in ruminants and serve as a potential source of energy for ruminant feeding [12]. For instance, Elseed et al. [13] reported that there was considerable variation among sorghum stalk varieties in terms of their chemical composition and relative chemical proportions. Similarly, Billa et al. [14] showed that sweet sorghum pith and bark fractions also exhibited substantial differences with respect to their composition, so that bark showed higher levels of total cellulose, hemicellulose, and lignin content, compared with pith, while pith was found to be twice as rich in sucrose and glucose, compared with bark. Specifically, sorghum is rich in various polyphenol compounds, and is characterized by high levels of 2,2-diphenyl-1-picrylhydrazyl (DPPH) free-radical scavenging capacity, leading to in vitro antioxidant activity [15]. Wang et al. [16] showed that sheep can effectively utilize nutrients in sorghum stalks, and reported higher nutrient degradability both in vitro and in vivo, indicating that sorghum stalks may offer high nutritive value to ruminants. Indeed, sorghum stalk, as a cheap crop, may be an ideal source of unconventional feed for ruminants. However, previous studies have analyzed total polyphenols using spectrophotometric methods; as a result, the subgroups of polyphenols are not yet fully understood [17,18], and the application of sorghum stalk remains restricted to some extent. Interestingly, polyphenol subgroups in sorghum stalks may be detected by high-performance liquid chromatography-tandem mass spectrometry (HPLC–MS) technology. We hypothesized that sorghum stalks would contain high levels of polyphenol compounds, exhibit high antioxidant activity, and improve ruminal fluid parameters in goats. Therefore, the aim of this study was to compare the chemical composition, polyphenol compounds, antioxidant activity, and in vitro incubation of different varieties of sorghum stalks with the ruminal fluid parameters of goats.

## 2. Materials and Methods

### 2.1. Plant Materials

**Figure 1:**
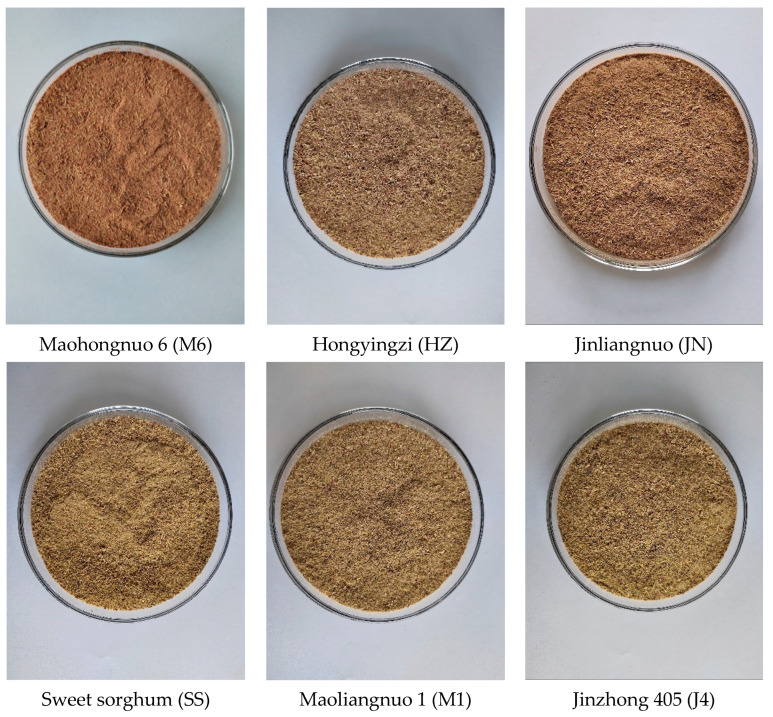
The pictures of different varieties of sorghum stalk grind samples.

Guizhou province is a major producer of Chinese liquor, and sorghum is the main raw material used for brewing. Because Guizhou province enjoys a subtropical monsoon climate, the local sorghums are particularly suitable for brewing products such as Moutai liquor. Sorghum stalks are a byproduct of the liquor industry, and these may be used as a source of unconventional feed for ruminants. For the present study, six widely available and commonly types of special sorghum (*Sorghum bicolor* L. Moench) varieties used for brewing liquor in Guizhou Province were selected. Accessions of sorghum stalk samples were obtained from the Guizhou University farm (Guiyang, China), and the scientific names of the sorghum varieties were as follows: maohongnuo 6 (M6), hongyingzi (HZ), jinliangnuo (JN), sweet sorghum (SS), maoliangnuo 1 (M1), and jinzhong 405 (J4). The main information descriptors, such as the varieties, origins, and abbreviations of sorghums, are summarized in Table 1. Six types of sorghum varieties were cultivated under the same conditions in a completely randomized design with three duplicates per sorghum variety. After sorghum was harvested at the yellow ripe stage, the sorghum stalk was cut (6–8 cm above the soil surface) using a cutting machine (TU43; Mitsubishi, Tokyo, Japan). After the seeds were harvested, the whole sorghum stalk was removed to the Institute of Animal Nutrition and Feed Laboratory (Guizhou University, China), and all samples were chopped into 2–3 cm lengths. Duplicates of sorghum stalk samples (each stalk was divided into 3 duplicates) were dried at 65 °C in a drying oven, and then smashed by a hammer-plate grinder (QE-200, Zhejiang Yili Industry and Trade Co., Ltd., Jinhua, China); each duplicate sorghum stalk (each sample had 3 duplicates) was then mixed and filtered through 80 mesh to prepare air-dried samples (n = 3); they were stored at 4 °C for further measurement. Pictures of ground sorghum stalk samples are shown in Figure 1.

### 2.2. Chemical Composition

Approximately 2.0 g of sorghum stalk was weighed, and dry matter (DM) was analyzed by drying in an electric vacuum drying oven at 105 °C for 2 h according to AOAC Method 930.15 [19]. Crude protein (CP) was analyzed by the Kjeldahl method, using automatic Kjeldahl apparatus (K1100, Hanon Technologies, Jinan, China). CP was calculated (Method 988.05) according to the following formula: total nitrogen × 6.25. Gross energy (GE) was analyzed using a bomb calorimeter with an O_2_ gas carrier (WGR-WR3, Changsha BENTE Instrument Co., Ltd., Changsha, China). Ash content was analyzed using a muffle furnace at 550 °C for 5 h (Method 942.05). Organic matter (OM) content was calculated as one hundred percent minus ash. Calcium (Ca) and phosphorus (P) were analyzed by the dry ash method (Method 927.02), and the photometric method (Method 965.17), respectively. Neutral detergent fiber (NDF), acid detergent fiber (ADF), and acid detergent lignin (ADL) were analyzed using an automatic fiber analyzer (Fibertherm FT 12, Gerhardt, Germany) according to the methods of Van Soest et al. [20]. All measurements were performed in triplicate. Hemicellulose and cellulose were calculated by trial and error methods, as follows: hemicellulose = NDF − ADF; cellulose = ADF − ADL. Total digestible nutrients (TDN), metabolizable energy (ME), and digestible energy (DE) were calculated according to the following equations, following Van Le et al. [21]: TDN (%) = 82.38 − (0.7515 × ADF); DE (Mcal/kg) = %TDN × 0.01 × 4.4); ME (MJ/kg) = (DE × 0.82) × 4.185.

### 2.3. Polyphenol Compounds

Subgroups of polyphenol compounds in sorghum stalks were analyzed according to the method of Wang et al. [22]. Briefly, approximately 0.5 g of sorghum stalk was weighed, added to 4 mL of 1% hydrochloric acid–methanol solution, mixed evenly, and then sonicated for 30 min. In addition, the mixture was centrifuged at 11,000× *g* for 30 min at 4 °C (TG-16G, Hunan Kaida Scientific Instrument Co., Ltd., Changsha, China) to prepare sorghum stalk extract for further analysis. Next, the supernatant was immediately transferred to a 0.22 μm nylon syringe, and the quantitative determination of polyphenol compounds was achieved using an HPLC–MS/MS machine (AGLIENT1260; Agilent Technologies Inc., Santa Clara, CA, USA) and a diode-array detector. The HPLC conditions were as follows: Agilent ZORBAX Eclipse Plus C18 column (3.5 µm × 2.1 mm × 150 mm); mobile phase A: acetonitrile; mobile phase B: 0.1% formic acid; injection temperature 4 °C, column temperature 35 °C; flow rate 0.3 mL/min; and injection volume 3 μL. A standard curve was established, and individual polyphenols were calculated according to the chromatogram peak area.

### 2.4. Antioxidant Activity

Antioxidant activity parameters for total antioxidant capacity (TAC; kit number A015-1-1), superoxide dismutase (SOD; kit number A001-1-1), glutathione peroxidase (GPX; kit number A005-1-1), catalase (CAT; kit number A007-1-1), and 2,2-diphenyl-1-picrylhydrazyl (DPPH) free-radical scavenging capacity (kit number A153-1-1) of the sorghum stalk extracts (as describe in Section 2.3 above) were analyzed according to the instructions of matched test kits provided by the Nanjing Jiancheng Bioengineering Institute (Nanjing, China).

### 2.5. In Vitro Gas Production

All experimental animal-care procedures were approved by the Rules of Animal Welfare and Experimental Animal Ethics of Guizhou University (Guiyang, China). Six healthy Qianbei brown goat does (Guizhou indigenous goat breed, Xishui, China) with similar body weights (42.50 ± 0.67 kg) were ruminal fluid donors. The goats were fed a concentrate/roughage ratio of 30:70 with a 13% CP total mixed ration according to the feeding standard of Qianbei brown goats (DB52/T 1377-2018). Before morning feeding, ruminal fluid was collected from animals using an ororuminal probe and the auto-suction pump (AP-9950; Tianjin Autoscience Instrument Co., Ltd., Tianjin, China), and the first 100 mL of ruminal fluid was discarded to avoid saliva. Ruminal fluid was stored in a 39 °C container, and immediately transferred to the in vitro rumen fermentation laboratory of the Institute of Animal Nutrition and Feed, Guizhou University. Next, the ruminal fluid was immediately passed through 4 layers of cheesecloth. Rumen fluid from the six goats was then mixed evenly in equal volume, to prepare an experimental ruminal fluid sample. In vitro gas production was detected using the procedure described by Menke and Steingass [23], as follows: (1) preparation of syringes: 0.50 g of each substrate was weighed by an analytical balance (with results correct to four decimal places), and removed to a 100 mL glass gas-tight syringe (Changzhou Mingyue Medical Equipment Co., Ltd., Changzhou, China). The syringe was incubated in a 39 °C water bath (SYG-2–8; Tianjin Taist Instrument Co., Ltd., Tianjin, China) after the plunger was greased with Vaseline. (2) Preparation of artificial saliva: buffer solution (35 g NaHCO_3_, 4 g NH_4_HCO_3_, and 1 L distilled water), macromineral solution (5.7 g Na_2_HPO_4_, 6.2 g KH_2_PO_4_, 0.6 g MgSO_4_, and 1 L distilled water), micromineral solution (13.2 g CaCl_2_·2H_2_O, 10.0 g MnCl_2_·4H_2_O, 1.0 g CoCl_2_·6H_2_O, 0.8 g FeCl_2_·6H_2_O, and 1 L distilled water), and resazurin aqueous solution (100 mg/100 mL) were added to a round flat-bottomed flask. (3) Detection of gas production: the ratio of artificial saliva:ruminal fluid was 2:1, and the solution was mixed well. Then, 30 mL of mixed solution was injected into a syringe, and the total gas production (GP) was read and calculated at 3, 6, 9, 12, 24, 48, 72, and 96 h. Each duplicate of sorghum stalk was incubated in two syringes, giving a total of six syringes per sample of in vitro gas production (n = 6). The GP was calculated using the method of Ørskov and McDonald [24], according to the following formula: y = a + b × (1 − e^−ct^), where y is the gas production volume at t h, a is the immediately soluble fraction, b is the insoluble fraction, c is the rate constant, t is the incubation time, and a + b is the potential extent for the GP. The organic matter digestibility (OMD), metabolizable energy (ME), and effective degradability (ED) were calculated using the following equations [25]: OMD (%) = 0.986 × GP (24 h) + 0.0606 × CP + 11.03; ME (MJ/kg) = −0.20 + 0.1410 × OMD; and ED (%) = a + bc/(k + c), where k = 0.031 h.

In addition, about 70% of the roughage nutrition disappeared within 24 h of in vitro gas production, indicating that it might ultimately all be degradable with this diet of ruminants [24]. For the purposes of the present study, then, rumen fermentation parameters were detected at 24 h incubation time. The syringe fermentation was stopped, and the pH value was detected immediately using a portable pH meter (pH 818, Guangdong, China). In addition, a fermentation:HCL (6 mol/L) ratio of 4:1 was prepared and mixed for further analysis of ammonia nitrogen (NH_3_-N) and volatile fatty acid (VFA). The ruminal fluid of NH_3_-N was detected using the steam distillation method described by Bremner and Keeney [26]. VFAs were detected using a Thermo TRACE 1310-ISQ gas chromatography–mass spectrum (GC–MS) machine (Thermo, Waltham, MA, USA). The GC conditions were as follows: chromatographic column: an Agilent HP-INNOWAX capillary column (30 m × 0.25 mm × 0.25 μm); injection temperature: 250 °C; ion power temperature: 230 °C; transmission line temperature: 250 °C; quadrupole temperature: 150 °C. Temperature programming was conducted as follows: the reaction temperature was initially 90 °C; it was then increased to 120 °C at 10 °C/min, then to 150 °C at 5 °C/min, and, finally, to 250 °C at 25 °C/min; this latter temperature was held for 2 min. Helium was used as the carrier gas, the rate was 1.0 mL/min; the injection volume was 1 μL, and the split ratio was 10:1. The MS conditions were as follows: electron impact ionization; ion energy of 70 eV; and use of the single ion monitoring scanning method. Individual VFAs consisted of acetic acid, propionic acid, and butyric acid, their sum was the total VFA (TVFA) concentration, and the ratio of acetic acid content to propionic acid content was the acetic: propionic ratio of the ruminal fluid.

### 2.6. Statistical Analysis

The sorghum stalk duplicate was set as the experimental unit for chemical composition, polyphenol compounds, and antioxidant activity parameters (n = 3); the syringe was set as the experimental unit for gas production kinetics, ruminal fluid fermentation, and GP parameters (n = 6). All data were analyzed using Statistical Analysis System software (Version 9.1.3; SAS Institute Inc., Cary, NC, USA). Data on sorghum-stalk chemical composition, polyphenol compounds, antioxidant activity, gas production kinetics, and ruminal fluid fermentation parameters were detected using one-way ANOVA: Y*_ij_* = µ + τ*_i_* + ε*_ij_*, where Y*_ij_* means observation, µ means overall mean, τ*_i_* means effect of the treatment, and ε*_ij_* means random error with a mean of 0 and variance σ^2^. Data on GP were obtained using two-factorial ANOVA: Y*_ijn_* = µ + S*_i_* + T*_j_* + (S*T)*_ij_* + ε*_ijn_*, where Y*_ijn_* means observation, µ means overall mean, S*_i_* means effect of sorghum stalk, T*_j_* means incubation time, (S*T)*_ij_* means effect of interaction between sorghum stalk and incubation time, and ε*_ijn_* means random error with mean 0 and variance σ^2^ [27]. The Pearson correlation coefficient (r) was used to determine the relationship between the total polyphenol content and antioxidant activity parameters in sorghum stalk extracts. Differences were considered to be statistically significant at the level of *p* < 0.05.

## 3. Results

### 3.1. Chemical Composition

No significant differences (*p* > 0.05) in GE were observed among the six varieties of sorghum stalks (Table 2). M1 contained a higher (*p* < 0.05) level of DM, and J4 contained a higher (*p* < 0.05) level of CP, relative to the other samples. In addition, M1 and J4 contained lower (*p* < 0.05) levels of OM and higher (*p* < 0.05) levels of ash, respectively. Significantly (*p* < 0.05) higher concentrations of NDF, ADF, and cellulose were observed in stalk JN, along with lower levels of TDN, DE, and ME. Stalk M6 had significantly (*p* < 0.05) higher levels of ADL, hemicellulose, Ca, and P.

### 3.2. Polyphenol Compounds

Protocatechualdehyde, epicatechin, vitexin-2-o-rhamnosid, rutin, isorhamnetin-3-o-neohespeidoside, hyperoside, hesperidin, resveratrol, psoralen, bergapten, asiatic acid, rhein, galangin, chrysophanol, and physcion were not detected among the six varieties of sorghum stalks (Table 3). Genistein was detected in M6, but not in any of the other five stalks. Among the samples, there were no significant differences (*p* > 0.05) in levels of gallic acid, nobiletin, or ursolic acid. Levels of chlorogenic acid, homoorientin, isovitexin, vitexin, rhoifolin, genistin, quercetin, apigenin, aloe emodin, emodin, and total polyphenols were significantly (*p* < 0.05) higher in M6 than in the other stalks. The HZ contained significantly higher (*p* < 0.05) levels of caffeic acid, kaempferol 3-rutinoside, and aurantio-obtusin; and lower (*p* < 0.05) levels of protocatechuic acid. Compared to the other stalks, JN contained a higher (*p* < 0.05) level of neohesperidin, naringin, and naringenin. M1 contained a higher (*p* < 0.05) level of tiliroside; however, its level of total polyphenols was lower (*p* < 0.05) than those of the other five stalks. The catechin and kaempferol contents in M1 were significantly (*p* < 0.05) higher than those in the other stalks. The level of umbelliferone in J4 was greater (*p* < 0.05) than that of the other sorghum stalks.

### 3.3. Antioxidant Activity Parameters

As shown in Table 4, stalk M6 showed higher (*p* < 0.05) levels of TAC, GPX, CAT, and DPPH free-radical scavenging capacity relative to the other five stalks. Similarly, compared to stalk JN, SS, and M1, stalk M6 contained a significantly higher level of SOD (*p* < 0.05).

### 3.4. Pearson Correlation Coefficients

There were significant (*p* < 0.05) positive correlations between total polyphenols and TAC, SOD, GPX, CAT, and DPPH free-radical scavenging capacity in sorghum stalk extracts (Table 5).

### 3.5. Total Gas Production

The GP was significantly (*p* < 0.05) influenced by both sorghum stalk varieties and incubation times (Table 6). However, there was no correlation (*p* = 0.0785) between sorghum stalk variety and incubation time with respect to GP.

### 3.6. Gas Production Kinetics

The stalk J4 displayed higher (*p* < 0.05) a and a + b values, relative to the other sorghum stalks (Table 7). Compared to the other five sorghum stalks, stalk M6 had a significantly lower (*p* < 0.05) b level, and stalk SS had a significantly higher (*p* < 0.05) c level. Additionally, stalk SS showed significantly higher (*p* < 0.05) levels of OMD and ME, and stalks SS and J4 had ED values that were significantly higher (*p* < 0.05) than those of M6 and JN.

### 3.7. Ruminal Fluid Fermentation Parameters

No significant differences (*p* > 0.05) were detected in the ruminal fluid with respect to pH, NH_3_-N, acetic acid, butyric acid, or TVFA concentrations (Table 8). In contrast, stalk M6 contained significantly higher (*p* < 0.05) levels of ruminal-fluid propionic acid; however, the ratio of acetic acid to propionic acid in its ruminal fluid was significantly lower (*p* < 0.05), compared with the other five varieties of sorghum stalks.

## 4. Discussion

The level of fiber is among the most important factors in feed that can affect the health and performance of ruminants [28]. Crude fibers are the main components of plant cell walls; these include cellulose, hemicellulose, and ADL, amongst others. In the context of the present study, it is of interest that the cell wall is associated with polyphenol compounds (such as p-coumaric and ferulic acids), and that p-Coumaric acid is the predominant p-hydroxycinnamic associated with the cell wall in sweet sorghum [14]. Hence, stalk M6 had high levels of total polyphenols, and thus contained lower levels of CP and higher levels of ADL. Elseed et al. [13] studied the chemical composition of sorghum stalk and found, depending on the varieties, that the proportions of CP, NDF, ADF, and hemicellulose in whole sorghum stalks ranged from 3.2–7.4%, 60.6–78.0%, 45.0–60.0%, and 4.2–18.5%, respectively. These findings are consistent with our results. In addition, Manea et al. [17] analyzed five varieties of sorghum stalks and found that their CP content was less than 2%; compared with this, our results indicated levels of CP which were two–three times higher. Agbagla-Dohnani et al. [29] showed that rice stalks exhibited great variability in chemical composition, such as DM, silica, ash, CP, NDF, ADF, and ADL. In addition, Firdous and Gilani [30] reported that plant maturity had a much greater impact on the chemical composition of whole sorghum plants, as well as leaf and stem fractions. Differences in chemical composition observed in other studies likely reflect differences between the parts of the plants used, and their maturity, in addition to soil, weather, and environmental characteristics. The data obtained in the present study correspond with the findings of Billa et al. [14], who indicated that different parts (bark and pith) of sweet sorghum showed variation in chemical composition.

Another previous study demonstrated that sorghum stalks contain many natural polyphenol compounds that show high antioxidant activity, and that their extracts can change bacterial morphology and internal structure, strongly inhibiting the growth of foodborne pathogens [31]. Similarly, Chen et al. [32] found that sweet sorghum stalk extract contains abundant levels of p-hydroxybenzoic acid, as well as caffeic, gentisic, chlorogenic, coumaric, and gallic acids, with potential antimicrobial effects and strong antioxidant properties. In addition, the authors of [33] reported that sorghum exhibits red, white, yellow, and brown colors, and these contain different totals of polyphenols. In the present study, the six varieties of sorghum stalks had different colors, resulting in different polyphenol compounds. We found that sorghum M6 was rich in 25 polyphenol compounds; sorghum HZ and JN were rich in 24 polyphenol compounds; and sorghums SS, M1, and J4 were rich in 23 polyphenol compounds. Sorghum stalk color is thus related to polyphenol content, and polyphenol content has also been shown to exhibit a strong positive correlation with DPPH free-radical scavenging capacity [15]. Using HPLC-ESI–MS/MS technology, Luo et al. [34] found that extracts of red sorghum bran contain abundant polyphenol compounds such as taxifolin, taxifolin hexoside, procyanidins, and epicatechin. In addition, Kalisz et al. [35] showed that red *Malinowy* rhubarb stalks exhibited an intense pink-red color, and also contained a high polyphenol content. Similarly, in the present study, we found that stalk M6 was red in color (Figure 1) and contained a higher level of total polyphenol compounds, suggesting that M6 may exhibit strong potential antioxidant and antimicrobial effects (e.g., against *Staphylococcus aureus* or *Escherichia coli*) [36].

Free radicals are byproducts of metabolism which influence the homeostasis between the generation and scavenging of radicals in vivo; this homeostasis mainly depends on the antioxidant system [37]. Moreover, free radicals are highly reactive molecules that bind and destroy body cells, and are the main cause of diseases and ageing [38]. Polyphenols are secondary metabolites characterized by one or more hydroxyl groups that bind to one or more aromatic rings; they are powerful antioxidants that complement and enhance the functions of antioxidant enzymes to protect against oxidative stress [39]. Additionally, polyphenols can achieve a high level of antioxidant activity in vitro by scavenging free radicals or limiting their formation [40]. Rodríguez-Muela et al. [41] showed that the inclusion of polyphenol-rich plants in the diet of lambs could improve plasma antioxidant activity parameters. In the present study, we found that stalk M6 contained a higher level of total polyphenols, and its extract showed higher antioxidant activity parameters, including TAC, SOD, GPX, CAT, and DPPH free-radical scavenging capacity. This could have occurred because (1) polyphenol compounds can improve antioxidant activity, to protect against oxidative stress in ruminants; (2) polyphenol compounds are oxidized by free radicals, resulting in the formation of less reactive and more stable molecules [42]; or (3) polyphenol compounds can chelate metal ions and form stable complexes with metal ions of the transition group and are thus involved in the antioxidant protection of the cell [7]. In line with our findings, Shih et al. [43] found that Moringa extract contained higher levels of polyphenol compounds, showed stronger hydrogen peroxide scavenging activity, and exhibited higher levels of SOD activity in vitro. However, chemical composition parameters varied widely among the six varieties of sorghum stalk, so that it could not be determined if antioxidant activity difference was responsible for polyphenols or chemical composition. This point needs to be addressed in future studies.

Polyphenol compounds are excellent natural antioxidants and free-radical scavengers that can eliminate free radicals in the bodies of animals [44]. Moreover, polyphenol compounds may also interact with iron and exhibit a stronger ability to scavenge free radicals in vitro [45]. Shen et al. [46] showed that polyphenol compounds exhibit scavenging activity with respect to strong superoxide radicals, hydroxyl radicals, as well as ferric reducing antioxidant power and moderate metal ion-chelating activity. Thus, significant positive correlations were observed between total polyphenols and TAC, SOD, GPX, CAT, and DPPH free-radical scavenging capacity. Our results are also in line with those reported by Kiselova et al. [47], who showed that the antioxidant capacity of plant extracts largely resulted from their polyphenol compounds, and that antioxidant activity and polyphenol content showed a stronger positive correlation. In short, our findings provide evidence that polyphenol compounds display higher levels of in vitro antioxidant activity. It is therefore reasonable to suggest that dietary supplementation with polyphenol-rich sorghum stalks might inhibit inflammatory reactions and improve the activities of antioxidants in goats; however, this needs to be validated by future in vivo experiments in ruminants.

In vitro gas production can reflect the type and degree of fermentation, and thereby serve as an important indicator for the nutritional value of feed [48]. Vasta et al. [49] showed that polyphenol compounds could inhibit ruminant methane production by inhibiting fibrolytic bacteria, decreasing fiber digestibility and H_2_ production, and reducing the protozoa population of the ruminal fluid. In the present study, M6 contained the lowest level of GP during the entire incubation period, probably because it contained higher levels of polyphenol compounds such as apigenin, genistin, vitexin, isovitexin, and aloe emodin, as well as higher total polyphenols; however, this assumption needs further validation. Nevertheless, our results are consistent those of with Lu et al. [18], who showed that high-polyphenol feed could inhibit methane production, and result in a low level of GP, in in vitro ruminal fluid incubation.

In the current study, J4 showed the highest a value, perhaps because it contained the highest level of CP. Recalling the suggestion by Tovar-Gomez et al. [50] that low levels of ADF and cellulose in crop stalks result in higher levels of b and c, we also note that, in the present study, stalk SS had higher c values. These differences might result from different levels of ADF and cellulose in sorghum stalks. In addition, Elseed et al. [13] showed that sorghum stalks exhibited lower degradability and lower potential feeding value when they contained a higher cell-wall content. In the current study, stalk M6 had low a, b, a + b and c values, perhaps due to its ADL content being higher than that of other sorghum stalks. Moreover, active substances, such as polyphenols in plants, may protect CP from degradation in the ruminal fluid of goats [51]. Thus, in the present study, SS had higher levels of OMD, ME, and ED, possibly because of its high chemical composition for TDN, DE, and ME values.

Polyphenol compounds could regulate ruminal-fluid ciliate and protozoal Gram-positive fibrolytic bacteria, leading to a reduction in VFA production, specifically for the decreased production of acetic acid [49]. Thus, polyphenols can influence gastrointestinal tract function and improve health in ruminants [52]. In addition, Odongo et al. [51] have shown that polyphenols can directly inhibit the growth of methanogens and hydrogen-producing microbes and decrease protozoal numbers, thereby decreasing ruminal-fluid CH_4_ production in ruminants. Interestingly, 3-(4-hydroxypheny1) propionic acid in ruminal fluid may result from the chemical reduction of dietary phenolic monomers by ruminal microorganisms [53]. Specifically, polyphenols could produce a positive fermentation pattern with a better ratio of acetic acid to propionic acid [54]. In the present study, we found that stalk M6 increased ruminal-fluid propionic acid and decreased the ratio of acetic acid to propionic acid. The possible reasons may be that (1) polyphenols are involved in propionic acid in ruminal fluid, possibly as the result of the interaction of polyphenols and the nonpolyphenolic polymer lignin [4]; (2) polyphenols destroy the integrity of the bacterial cell membrane structure, resulting in the exudation of intracellular molecules and an increase in the electrolyte content in the cell culture medium [31]; and (3) polyphenols could increase microbial protein flow from the rumen and increase the efficiency of substrate utilization [54]. In short, because stalk M6 contained an abundance of polyphenols, this suggests that the propionic acid ratio of the ruminal fluid was increased, and that methane emissions were effectively suppressed. These findings are similar to those reported by Wang et al. [55], who showed that the addition of polyphenol-rich *Castanea mollissima* Blume could significantly increase the propionic acid content and reduce the acetic acid/propionic acid ratio in in vitro rumen fermentation. However, further experimental data from in vivo feeding trials with ruminants are required to validate these findings.

## 5. Conclusions

The results of the present study indicate that the chemical composition, polyphenol compounds, antioxidant activity, and in vitro rumen fermentation differed greatly among six varieties of sorghum stalks. Specifically, stalk M6 showed higher levels of total polyphenols, antioxidant activity parameters, and propionic acid, with lower levels of GP and a lower ratio of acetic acid to propionic acid. Further research is needed involving in vivo feeding trials with ruminants to analyze how polyphenol compounds from sorghum stalks affect microorganisms and antioxidant potential in ruminal fluid.

## Figures and Tables

**Table 1:** The information for six varieties of sorghums in this study.

Number	Sorghum Varieties	Origin	Abbreviation
I	Maohongnuo 6	China	M6
II	Hongyingzi	China	HZ
III	Jinliangnuo	China	JN
IV	Sweet sorghum	China	SS
V	Maoliangnuo 1	China	M1
VI	Jinzhong 405	China	J4

**Table 2:** Comparison of chemical composition of six varieties of sorghum stalks.

Items	Varieties						SEM	*p*-Value
	M6	HZ	JN	SS	M1	J4		
DM, %	28.64 ^b^	28.32 ^c^	28.69 ^b^	19.11 ^e^	29.20 ^a^	25.29 ^d^	0.0361	<0.0001
CP, % of DM	3.78 ^e^	5.58 ^c^	5.09 ^d^	5.56 ^c^	5.99 ^b^	7.15 ^a^	0.0607	<0.0001
GE, MJ/kg of DM	16.83	17.33	17.95	17.60	17.73	17.82	0.3495	0.3513
OM, % of DM	94.02 ^c^	94.04 ^c^	94.72 ^b^	95.51 ^a^	93.61 ^d^	93.53 ^d^	0.0382	<0.0001
Ash, % of DM	5.98 ^b^	5.96 ^b^	5.28 ^c^	4.49 ^d^	6.39 ^a^	6.47 ^a^	0.0386	<0.0001
NDF, % of DM	71.62 ^b^	68.14 ^c^	73.21 ^a^	50.87 ^f^	60.50 ^e^	62.15 ^d^	0.4013	<0.0001
ADF, % of DM	45.65 ^b^	42.01 ^c^	49.41 ^a^	31.35 ^f^	36.32 ^e^	37.64 ^d^	0.2024	<0.0001
ADL, % of DM	8.08 ^a^	6.31 ^c^	6.96 ^b^	3.95 ^f^	4.96 ^e^	5.41 ^d^	0.0838	<0.0001
Hemicellulose, % of DM	25.97 ^a^	26.14 ^a^	23.80 ^b^	19.52 ^c^	24.18 ^b^	24.51 ^b^	0.3087	<0.0001
Cellulose, % of DM	37.57 ^b^	35.69 ^c^	42.45 ^a^	27.40 ^f^	31.36 ^e^	32.23 ^d^	0.1833	<0.0001
Ca, % of DM	1.16 ^a^	0.58 ^b^	0.53 ^b^	0.57 ^b^	0.67 ^b^	0.67 ^b^	0.1142	0.0485
P, % of DM	0.20 ^a^	0.13 ^bc^	0.18 ^ab^	0.13 ^bc^	0.07 ^d^	0.09 ^dc^	0.0139	0.0014
TDN, % of DM	48.08 ^e^	50.81 ^d^	45.25 ^f^	58.82 ^a^	55.09 ^b^	54.09 ^c^	0.1524	<0.0001
DE, Mcal/kg	2.12 ^e^	2.24 ^d^	1.99 ^f^	2.59 ^a^	2.42 ^b^	2.38 ^c^	0.0067	<0.0001
ME, MJ/kg	7.26 ^e^	7.67 ^d^	6.83 ^f^	8.88 ^a^	8.32 ^b^	8.17 ^c^	0.0230	<0.0001

Values with the same superscript letters in the same line are of no significant difference (*p* > 0.05); those with different letters are of significant difference (*p* < 0.05). M6, maohongnuo 6; HZ, hongyingzi; JN, jinliangnuo; SS, sweet sorghum; M1, maoliangnuo 1; J4, jinzhong 405; DM, dry matter; CP, crude protein; GE, gross energy; OM, organic matter; NDF, neutral detergent fiber; ADF, acid detergent fiber; ADL, acid detergent lignin; Ca, calcium; P, phosphorus; TDN, total digestible nutrient; ME, metabolizable energy; DE, digestible energy.

**Table 3:** Comparison of polyphenol compounds of six varieties of sorghum stalks.

Items, ng/g	Varieties						SEM	*p*-Value
	M6	HZ	JN	SS	M1	J4		
Gallic acid	37.04	30.56	33.00	36.19	32.68	36.31	1.9178	0.3213
Protocatechuic acid	356.25 ^cd^	318.64 ^d^	317.67 ^d^	450.13 ^ab^	398.15 ^bc^	477.08 ^a^	19.0688	0.0028
Protocatechualdehyde	nd	nd	nd	nd	nd	nd	-	-
Chlorogenic acid	157.43 ^a^	95.95 ^b^	45.22 ^d^	54.87 ^cd^	60.22 ^cd^	74.77 ^bc^	5.9469	<0.0001
Catechin	18.01 ^c^	31.65 ^b^	17.55 ^c^	21.52 ^bc^	46.42 ^a^	26.38 ^bc^	2.9806	0.0032
Caffeic acid	270.64 ^bc^	341.14 ^a^	164.13 ^d^	260.42 ^c^	239.26 ^c^	309.66 ^ab^	10.6066	0.0001
Epicatechin	nd	nd	nd	nd	nd	nd	-	-
Homoorientin	354.18 ^a^	292.93 ^b^	148.67 ^d^	185.45 ^d^	313.60 ^ab^	237.44 ^c^	13.0793	<0.0001
Vitexin-2-o-rhamnosid	nd	nd	nd	nd	nd	nd	-	-
Rutin	nd	nd	nd	nd	nd	nd	-	-
Neohesperidin	12.52 ^b^	13.73 ^b^	36.33 ^a^	10.03 ^b^	14.86 ^b^	11.39 ^b^	2.2072	0.0009
Isorhamnetin-3-o-neohespeidoside	nd	nd	nd	nd	nd	nd	-	-
Isovitexin	1072.89 ^a^	192.14 ^d^	397.67 ^b^	151.45 ^d^	172.96 ^d^	234.49 ^c^	10.7116	<0.0001
Vitexin	1089.96 ^a^	196.60 ^d^	418.07 ^b^	177.68 ^d^	185.70 ^d^	274.38 ^c^	11.2289	<0.0001
Hyperoside	nd	nd	nd	nd	nd	nd	-	-
Kaempferol 3-rutinoside	23.30 ^c^	78.70 ^a^	20.75 ^c^	59.55 ^ab^	32.30 ^c^	56.55 ^b^	5.5120	0.0011
Rhoifolin	347.25 ^a^	96.14 ^bc^	78.77 ^c^	95.20 ^bc^	80.97 ^c^	129.83 ^b^	11.1883	<0.0001
Naringin	107.36 ^ab^	74.02 ^bc^	127.33 ^a^	47.53 ^c^	46.81 ^c^	52.29 ^c^	9.4232	0.0028
Hesperidin	nd	nd	nd	nd	nd	nd	-	-
Genistin	6588.96 ^a^	1136.61 ^b^	941.66 ^bc^	837.33 ^c^	1033.64 ^bc^	940.99 ^bc^	55.2716	<0.0001
Umbelliferone	23.62 ^bc^	38.75 ^ab^	18.04 ^c^	18.45 ^c^	30.25 ^bc^	47.39 ^a^	4.0702	0.0086
Tiliroside	17.59 ^c^	43.15 ^ab^	23.56 ^bc^	55.99 ^a^	26.29 ^bc^	37.62 ^abc^	5.0550	0.0133
Resveratrol	nd	nd	nd	nd	nd	nd	-	-
Quercetin	8.23 ^a^	6.15 ^b^	5.05 ^b^	4.67 ^b^	4.41 ^b^	8.81 ^a^	0.4364	0.0006
Apigenin	20462.63 ^a^	2476.39 ^b^	2239.26 ^b^	1008.49 ^c^	1481.75 ^c^	2693.96 ^b^	166.8909	<0.0001
Naringenin	397.06 ^b^	335.71 ^c^	473.37 ^a^	163.80 ^e^	255.09 ^d^	245.85 ^d^	14.3457	<0.0001
Genistein	76.88	nd	nd	nd	nd	nd	-	-
Kaempferol	54.87 ^c^	40.93 ^c^	36.01 ^c^	410.56 ^b^	563.29 ^a^	419.53 ^b^	22.2789	<0.0001
Psoralen	nd	nd	nd	nd	nd	nd	-	-
Aurantio-obtusin	17.98 ^b^	43.56 ^a^	25.37 ^b^	12.49 ^b^	20.68 ^b^	16.23 ^b^	4.1026	0.0215
Bergapten	nd	nd	nd	nd	nd	nd	-	-
Asiatic acid	nd	nd	nd	nd	nd	nd	-	-
Rhein	nd	nd	nd	nd	nd	nd	-	-
Aloe emodin	320.27 ^a^	87.92 ^b^	71.81 ^bc^	32.28 ^c^	53.84 ^bc^	58.92 ^bc^	11.7452	<0.0001
Nobiletin	5.43	6.74	8.12	6.04	6.97	6.45	0.8341	0.5113
Galangin	nd	nd	nd	nd	nd	nd	-	-
Emodin	24.95 ^a^	14.33 ^c^	15.95 ^b^	nd	nd	nd	0.3292	<0.0001
Chrysophanol	nd	nd	nd	nd	nd	nd	-	-
Physcion	nd	nd	nd	nd	nd	nd	-	-
Ursolic acid	2.44	3.16	2.72	2.37	2.36	2.31	0.2598	0.4200
Total polyphenols	31847.74 ^a^	5995.59 ^b^	5666.09 ^b^	4102.51 ^d^	5102.50 ^c^	6398.64 ^b^	215.0871	<0.0001

Values with the same superscript letters in the same line are of no significant difference (*p* > 0.05); those with different letters are of significant difference (*p* < 0.05). M6, maohongnuo 6; HZ, hongyingzi; JN, jinliangnuo; SS, sweet sorghum; M1, maoliangnuo 1; J4, jinzhong 405. nd, not detected.

**Table 4:** Comparison of antioxidant activity parameters of six varieties of sorghum stalks.

Items	Varieties						SEM	*p*-Value
	M6	HZ	JN	SS	M1	J4		
TAC, U/mL	13.22 ^a^	7.42 ^b^	7.08 ^b^	6.59 ^b^	6.88 ^b^	7.42 ^b^	0.4245	<0.0001
SOD, U/mL	168.05 ^a^	159.06 ^ab^	153.75 ^b^	123.48 ^d^	140.92 ^c^	162.06 ^ab^	3.9206	<0.0001
GPX, U/mL	92.32 ^a^	80.27 ^b^	82.92 ^b^	68.61 ^d^	74.88 ^c^	81.89 ^b^	1.6207	<0.0001
CAT, U/mL	35.41 ^a^	27.44 ^bc^	26.74 ^bc^	24.34 ^c^	26.61 ^bc^	30.38 ^b^	1.2326	0.0006
DPPH scavenging capacity, %	85.65 ^a^	75.10 ^b^	73.04 ^bc^	62.97 ^d^	68.27 ^c^	73.48 ^bc^	1.6999	<0.0001

Values with the same superscript letters in the same line are of no significant difference (*p* > 0.05); those with different letters are of significant difference (*p* < 0.05). M6, maohongnuo 6; HZ, hongyingzi; JN, jinliangnuo; SS, sweet sorghum; M1, maoliangnuo 1; J4, jinzhong 405; TAC, total antioxidant capacity; SOD, superoxide dismutase; GPX, glutathione peroxidase; CAT, catalase; DPPH, 2,2-diphenyl-1-picrylhydrazyl free-radical scavenging capacity.

**Table 5:** Pearson correlation coefficients between total polyphenols and antioxidant activity parameters.

Items		TAC	SOD	GPX	CAT	DPPH Scavenging Capacity
Total polyphenols	r	0.968	0.530	0.750	0.814	0.805
	*p*	<0.0001	0.0237	0.0003	<0.0001	<0.0001

TAC, total antioxidant capacity; SOD, superoxide dismutase; GPX, glutathione peroxidase; CAT, catalase; DPPH, 2,2-diphenyl-1-picrylhydrazyl free-radical scavenging capacity.

**Table 6:** Comparison of total gas production of six varieties of sorghum stalks.

Varieties, mL	Incubation Time, h									SEM	*p*-Value		
	3	6	9	12	24	36	48	72	96		Stalk	Time	S × T
M6	7.33	12.67	17.00	22.33	41.67	52.33	60.50	67.67	70.00	3.6796	<0.0001	<0.0001	0.0785
HZ	12.17	20.00	25.00	29.50	50.83	62.50	70.17	76.83	79.83				
JN	9.17	16.17	20.83	25.83	46.50	57.83	68.50	79.83	83.50				
SS	9.17	34.33	41.50	47.33	59.83	75.83	81.17	87.33	88.67				
M1	7.67	22.17	27.50	33.33	53.67	66.83	71.00	75.33	80.33				
J4	7.33	20.33	25.83	32.00	51.83	67.83	79.33	86.50	89.67				

Stalk, effect of sorghum stalk; time, effect of incubation time; S × T, effect of sorghum stalk and incubation time interactions. M6, maohongnuo 6; HZ, hongyingzi; JN, jinliangnuo; SS, sweet sorghum; M1, maoliangnuo 1; J4, jinzhong 405.

**Table 7:** Comparison of in vitro gas production kinetics of six varieties of sorghum stalks.

Items	Varieties						SEM	*p*-Value
	M6	HZ	JN	SS	M1	J4		
a, mL	3.97 ^c^	5.08 ^b^	2.91 ^c^	3.75 ^c^	3.62 ^c^	6.25 ^a^	0.3201	<0.0001
b, mL	73.24 ^b^	86.18 ^a^	88.83 ^a^	89.05 ^a^	85.25 ^a^	97.16 ^a^	3.8328	0.0157
a + b, mL	77.20 ^c^	91.27 ^ab^	91.74 ^ab^	92.80 ^ab^	88.88 ^bc^	103.41 ^a^	3.8099	0.0074
c, % h	0.035 ^b^	0.036 ^b^	0.029 ^b^	0.057 ^a^	0.042 ^ab^	0.036 ^b^	0.0049	0.0114
OMD, %	52.34 ^c^	61.49 ^abc^	57.19 ^bc^	70.36 ^a^	64.31 ^ab^	62.57 ^abc^	3.5647	0.0263
ME, MJ/kg	7.18 ^c^	8.47 ^abc^	7.86 ^bc^	9.72 ^a^	8.87 ^ab^	8.62 ^abc^	0.5026	0.0263
ED, %	42.58 ^c^	50.70 ^abc^	45.75 ^bc^	59.26 ^a^	52.01 ^ab^	58.10 ^a^	2.7503	0.0019

Values with the same superscript letters in the same line are of no significant difference (*p* > 0.05); those with different letters are of significant difference (*p* < 0.05). a, immediately soluble fraction; b, insoluble fraction; a + b, potential extent of gas production; c, rate constant; OMD, organic matter digestibility; ME, metabolizable energy; ED, effective degradability. M6, maohongnuo 6; HZ, hongyingzi; JN, jinliangnuo; SS, sweet sorghum; M1, maoliangnuo 1; J4, jinzhong 405.

**Table 8:** Comparison of rumen fermentation parameters of six varieties of sorghum stalks at 24 h.

Items	Varieties						SEM	*p*-Value
	M6	HZ	JN	SS	M1	J4		
pH	6.42	6.38	6.36	6.22	6.23	6.28	0.0873	0.3296
NH_3_-N, mg/dL	5.95	5.90	6.10	6.94	6.92	6.98	0.3158	0.0649
Acetic acid, mmol/L	62.55	66.35	66.67	66.26	66.22	64.49	1.4283	0.3421
Propionic acid, mmol/L	21.87 ^a^	17.12 ^b^	17.41 ^b^	16.86 ^b^	16.77 ^b^	18.61 ^b^	0.6622	0.0011
Butyric acid, mmol/L	4.42	5.57	5.32	5.55	5.75	5.55	0.4155	0.3176
TVFA, mmol/L	88.18	89.04	89.74	88.67	88.73	88.64	1.5106	0.9853
Acetic acid to propionic acid ratio	2.87 ^b^	3.88 ^a^	3.84 ^a^	3.94 ^a^	3.97 ^a^	3.47 ^a^	0.1586	0.0025

Values with the same superscript letters in the same line are of no significant difference (*p* > 0.05); those with different letters are of significant difference (*p* < 0.05). M6, maohongnuo 6; HZ, hongyingzi; JN, jinliangnuo; SS, sweet sorghum; M1, maoliangnuo 1; J4, jinzhong 405; NH_3_-N, ammonia nitrogen; TVFA, total volatile fatty acid.

## Data Availability

Data are contained within the article.

## References

[1] Shen N., Wang T., Gan Q., Liu S., Wang L., Jin B. (2022). Plant flavonoids: Classification, distribution, biosynthesis, and antioxidant activity. Food Chem..

[2] Campos M.R.S., Vuolo M.M., Lima V.S., Junior M.R.M. (2019). Bioactive compounds: Health benefits and potential applications. Phenolic Compounds: Structure, Classification, and Antioxidant Power.

[3] Bešlo D., Golubić N., Rastija V., Agić D., Karnaš M., Šubarić D., Lučić B. (2023). Antioxidant activity, metabolism, and bioavailability of polyphenols in the diet of animals. Antioxidants.

[4] Aderao G.N., Sahoo A., Bhatt R.S., Kumawat P.K., Soni L. (2018). In vitro rumen fermentation kinetics, metabolite production, methane and substrate degradability of polyphenol rich plant leaves and their component complete feed blocks. J. Anim. Sci. Technol..

[5] Cattani M., Tagliapietra F., Bailoni L., Schiavon S. (2012). Synthetic and natural polyphenols with antioxidant properties stimulate rumen microbial growth in vitro. Anim. Prod. Sci..

[6] Uushona T., Chikwanha O.C., Katiyatiya C.L.F., Tayengwa T., Strydom P.E., Mapiye C. (2022). Ruminant meat production and quality enhancement, nematode suppression and greenhouse gas emission mitigation: A sustainable paradigm for valorisation of acacia leaves. Anim. Feed Sci. Tech..

[7] Bešlo D., Došlic G., Agic D., Rastija V., Šperanda M., Gantner V., Lucic B. (2022). Polyphenols in ruminant nutrition and their effects on reproduction. Antioxidants.

[8] Ramos M., Laveriano E., Sebastián L.S., Perez M., Jiménez A., Lamuela-Raventos R.M., Garrigós M.C., Vallverdú-Queralt A. (2023). Rice straw as a valuable source of cellulose and polyphenols: Applications in the food industry. Trends Food Sci. Tech..

[9] Elzaawely A.A., Maswada H.F., El-Sayed M.E.A., Ahmed M.E. (2017). Phenolic compounds and antioxidant activity of rice straw extract. Int. Lett. Nat. Sci..

[10] Li J., Ouyang Z., Liu P., Zhao X., Wu R., Zhang C., Lin C., Li Y., Guo X. (2021). Distribution and characteristics of microplastics in the basin of Chishui River in Renhuai, China. Sci. Total Environ..

[11] Kangama C.O., Xu R.M. (2005). Introduction of sorghum (*Sorghum bicolor* (L.) Moench) into China. Afr. J. Biotechnol..

[12] Cao C., Yang Z., Han L., Jiang X., Ji G. (2015). Study on in situ analysis of cellulose, hemicelluloses and lignin distribution linked to tissue structure of crop stalk internodal transverse section based on FTIR microspectroscopic imaging. Cellulose.

[13] Elseed A.M.A.F., Eldaim N.I.N., Amasaib E.O. (2007). Chemical composition and in situ dry matter degradability of stover fractions of five sorghum varieties. J. Appl. Sci. Res..

[14] Billa E., Koullas D.P., Monties B., Koukios E.G. (1997). Structure and composition of sweet sorghum stalk components. Ind. Crop. Prod..

[15] Choi S.C., Kim J.M., Lee Y.G., Kim C. (2019). Antioxidant activity and contents of total phenolic compounds and anthocyanins according to grain colour in several varieties of *Sorghum bicolor* (L.) moench. Cereal Res. Commun..

[16] Wang J., Zhang Z., Liu H., Xu J., Liu T., Wang C., Zheng C. (2022). Evaluation of gas production, fermentation parameters, and nutrient degradability in different proportions of sorghum straw and ammoniated wheat straw. Fermentation.

[17] Manea V., Tanase A., Casarica A., Albulescu R., Radulescu G., Campeanu G., Israel-Roming F., Stoina G. (2010). Study of the chemical composition of sweet sorghum stalks depleted in carbohydrates with applications in obtaining bioethanol. Analele Ştiinţifice AleUniv. Alexandru Ioan Cuza Secţiunea Genet. Şi Biol. Mol..

[18] Lu Q., Luo Q., Li J., Wang X., Ban C., Qin J., Tian Y., Tian X.Z., Chen X. (2022). Evaluation of the chemical composition, bioactive substance, gas production, and rumen fermentation parameters of four types of distiller’s grains. Molecules.

[19] AOAC (2005). Official Methods of Analysis.

[20] Van Soest P.V., Robertson J.B., Lewis B.A. (1991). Methods for dietary fiber, neutral detergent fiber, and nonstarch polysaccharides in relation to animal nutrition. J. Dairy Sci..

[21] Van Le H., Nguyen D.V., Vu Nguyen Q., Malau-Aduli B.S., Nichols P.D., Malau-Aduli A.E.O. (2019). Fatty acid profiles of muscle, liver, heart and kidney of Australian prime lambs fed different polyunsaturated fatty acids enriched pellets in a feedlot system. Sci. Rep..

[22] Wang X., Li J.X., Zhou D., Qin J.X., Xu Y.Q., Lu Q., Tian X.Z. (2024). Effects of *Rosa roxburghii* tratt seed on the growth performance, meat quality, and sensory evaluation characteristics in growing rabbits. Meat Sci..

[23] Menke K.H., Steingass H. (1988). Estimation of the energetic feed value obtained from chemical analysis and in vitro gas production using rumen fluid. Anim. Res. Dev..

[24] Ørskov E.R., McDonald I. (1979). The estimation of protein degradability in the rumen from incubation measurements weighted according to rate of passage. J. Agr. Sci..

[25] Tian X.Z., Paengkoum P., Paengkoum S., Thongpe S., Ban C. (2018). Comparison of forage yield, silage fermentative quality, anthocyanin stability, antioxidant activity, and in vitro rumen fermentation of anthocyanin-rich purple corn (*Zea mays* L.) stover and sticky corn stover. J. Integr. Agr..

[26] Bremner J.M., Keeney D.R. (1965). Steam distillation methods for determination of ammonium, nitrate and nitrite. Anal. Chim. Acta.

[27] Kaps M., Lamberson W.R. (2004). Biostatistics for Animal Science.

[28] Waghorn G.C., Clark D.A. (2004). Feeding value of pastures for ruminants. N. Zeal. Vet. J..

[29] Agbagla-Dohnani A., Nozière P., Clément G., Doreau M. (2001). In sacco degradability, chemical and morphological composition of 15 varieties of European rice straw. Anim. Feed Sci. Tech..

[30] Firdous R., Gilani A.H. (2001). Changes in chemical composition of sorghum as influenced by growth stage and cultivar. Asian Austral. J. Anim..

[31] Chen H., Xu Y., Chen H., Liu H., Yu Q., Han L. (2022). Isolation and identification of polyphenols from fresh sweet sorghum stems and their antibacterial mechanism against foodborne pathogens. Front. Bioeng. Biotech..

[32] Chen H., Tian X., Yu Q., Hu W., Chen J., Zhou L. (2021). Sweet sorghum stalks extract has antimicrobial activity. Ind. Crops Prod..

[33] Rhodes D.H., Stephen K. (2016). Sorghum (*Sorghum bicolor* (L.) Moench) genotypes with contrasting polyphenol compositions differentially modulate inflammatory cytokines in mouse macrophages. J. Chem..

[34] Luo X., Cui J., Zhang H., Duan Y., Zhang D., Cai M., Chen G. (2018). Ultrasound assisted extraction of polyphenolic compounds from red sorghum (*Sorghum bicolor* L.) bran and their biological activities and polyphenolic compositions. Ind. Crops Prod..

[35] Kalisz S., Oszmiański K., Kolniak-Ostek J., Grobelna A., Kieliszek M., Cendrowski A. (2020). Effect of a variety of polyphenols compounds and antioxidant properties of rhubarb (*Rheum rhabarbarum*). LWT—Food Sci. Technol..

[36] Martelli G., Giacomini D. (2008). Antibacterial and antioxidant activities for natural and synthetic dual-active compounds. Eur. J. Med. Chem..

[37] Warraich U.E.A., Hussain F., Kayani H.U.R. (2020). Aging-oxidative stress, antioxidants and computational modeling. Heliyon.

[38] Andrés C.M.C., de la Lastra J.M.P., Juan C.A., Plou F.J., Pérez-Lebeña E. (2023). From reactive species to disease development: Effect of oxidants and antioxidants on the cellular biomarkers. J. Biochem. Mol. Toxic..

[39] Stagos D. (2020). Antioxidant activity of polyphenolic plant extracts. Antioxidants.

[40] Chiva-Blanch G., Visioli F. (2012). Polyphenols and health: Moving beyond antioxidants. J. Berry Res..

[41] Rodríguez-Muela C., Rodríguez H.E., Arzola C., Díaz-Plascencia D., Ramírez-Godínez J.A., Flores-Mariñelarena A., Mancillas-Flores P.F., Corral G. (2015). Antioxidant activity in plasma and rumen papillae development in lambs fed fermented apple pomace. J. Anim. Sci..

[42] Mathew S., Abraham T.E., Zakaria Z.A. (2015). Reactivity of phenolic compounds towards free radicals under in vitro conditions. J. Food Sci. Tech..

[43] Shih M.C., Chang C.M., Kang S.M., Tsai M.L. (2011). Effect of different parts (leaf, stem and stalk) and seasons (summer and winter) on the chemical compositions and antioxidant activity of Moringa oleifera. Int. J. Mol. Sci..

[44] Avila-Nava A., Medina-Vera I., Toledo-Alvarado H., Corona L., Márquez-Mota C.C. (2023). Supplementation with antioxidants and phenolic compounds in ruminant feeding and its effect on dairy products: A systematic review. J. Dairy Res..

[45] Perron N.R., Brumaghim J.L. (2009). A review of the antioxidant mechanisms of polyphenol compounds related to iron binding. Cell Biochem. Biophys..

[46] Shen Y., Zhang H., Cheng L., Wang L., Qian H., Qi X. (2016). In Vitro and in vivo antioxidant activity of polyphenols extracted from black highland barley. Food Chem..

[47] Kiselova Y., Ivanova D., Chervenkov T., Gerova D., Yankova T. (2010). Correlation between the in vitro antioxidant activity and polyphenol content of aqueous extracts from Bulgarian herbs. Phytother. Res..

[48] Sallam S.M.A., Nasser M.E.A., El-Waziry A.M., Bueno I.C.S., Abdalla A.L. (2007). Use of an in vitro rumen gas production technique to evaluate some ruminant feedstuffs. J. Appl. Sci. Res..

[49] Vasta V., Daghio M., Cappucci A., Buccioni A., Serra A., Viti C., Mele M. (2019). Invited review: Plant polyphenols and rumen microbiota responsible for fatty acid biohydrogenation, fiber digestion, and methane emission: Experimental evidence and methodological approaches. J. Dairy Sci..

[50] Tovar-Gomez M.R., Emile J.C., MichaletDoreau B., Barriere Y. (1997). In situ degradation kinetics of maize hybrid stalks. Anim. Feed Sci. Technol..

[51] Odongo N.E., Garcia M., Viljoen G.J. (2010). Sustainable Improvement of Animal Production and Health.

[52] Theodorou M.K., Kingston-Smith A.H., Winters A.L., Lee M.R.F., Minchin F.R., Morris P., MacRae J. (2006). Polyphenols and their influence on gut function and health in ruminants: A review. Environ. Chem. Lett..

[53] Cremin J.D., Drackley J.K., Grum D.E., Hansen L.R., Fahey G.C. (1994). Effects of reduced phenolic acids on metabolism of propionate and palmitate in bovine liver tissue in vitro. J. Dairy Sci..

[54] Parmar P., Bhatt S., Dhyani S., Jain A. (2012). Phytochemical studies of the secondary metabolites of *Ziziphus mauritania* Lam. Leaves. Int. J. Curr. Pharm. Res..

[55] Wang Y., Yu S., Li Y., Zhang S., Qi X., Guo K., Guo Y., Fortina R. (2021). Pilot study of the effects of polyphenols from chestnut involucre on methane production, volatile fatty acids, and ammonia concentration during in vitro rumen fermentation. Animals.

